# DIABETES AS A RISK FACTOR FOR MILD COGNITIVE IMPAIRMENT IN OLDER RESIDENTS OF RURAL WEST TEXAS

**DOI:** 10.1093/geroni/igz038.3160

**Published:** 2019-11-08

**Authors:** Gabriela Arandia, Annette Boles, Veronica Lopez, Volker Neugebauer

**Affiliations:** Texas Tech University Health Sciences Center - Garrison Institute on Aging, Lubbock, Texas, United States

## Abstract

While a growing body of evidence suggests a link between diabetes and Alzheimer’s disease, few studies have examined the impact of diabetes on mild cognitive impairment, the precursor to Alzheimer’s disease, especially among older, rural, and ethnically diverse populations. Using data from Project FRONTIER (Facing Rural Obstacles to Healthcare Now Through Intervention, Education, & Research), a longitudinal cohort aging study in rural West Texas, the aim of this study was to compare the risk for mild cognitive impairment among participants who, according to blood sugar levels, were pre-diabetic/diabetic versus normal. This study uses baseline and 3-year follow-up data from a subsample (recruited from Cochran County) of the larger, four-county sample of Project FRONTIER. The study sample (n=206) ranged from 40 to 87 years old (mean age: 58.3 + 11.7 years old), was predominantly female (73.3%), White (88.4%), with slightly over half self-reporting as Hispanic (51.0%). Logistic regression results revealed that those who had prediabetes/diabetes had 1.81 times the risk for developing mild cognitive impairment compared to those who had normal blood sugar levels. These findings indicate the need for earlier intervention for improved diabetes prevention, self-management, and control (diet, physical activity, treatments) to help offset the development of mild cognitive impairment, which could progress to Alzheimer’s disease later in life. More research is needed to confirm the link between pre-diabetes/diabetes and mild cognitive impairment in other populations and settings.

